# 2-Cell-like Cells: An Avenue for Improving SCNT Efficiency

**DOI:** 10.3390/biom12111611

**Published:** 2022-11-01

**Authors:** Bo Fu, Hong Ma, Di Liu

**Affiliations:** 1 Institute of Animal Husbandry, Heilongjiang Academy of Agricultural Sciences, Harbin 150086, China; 2 Key Laboratory of Combining Farming and Animal Husbandry, Ministry of Agriculture and Rural Affairs, Harbin 150086, China

**Keywords:** 2-cell-like cells, totipotency, preimplantation embryo, somatic cell nuclear transfer

## Abstract

After fertilization, the zygote genome undergoes dramatic structural reorganization to ensure the establishment of totipotency, and then the totipotent potential of the zygote or 2-cell-stage embryo progressively declines. However, cellular potency is not always a one-way street. Specifically, a small number of embryonic stem cells (ESCs) occasionally overcome epigenetic barriers and transiently convert to a totipotent status. Despite the significant potential of the somatic cell nuclear transfer (SCNT) technique, the establishment of totipotency is often deficient in cloned embryos. Because of this phenomenon, the question arises as to whether strategies attempting to induce 2-cell-like cells (2CLCs) can provide practical applications, such as reprogramming of somatic cell nuclei. Inspired by strategies that convert ESCs into 2CLCs, we hypothesized that there will be a similar pathway by which cloned embryos can establish totipotent status after SCNT. In this review, we provide a snapshot of the practical strategies utilized to induce 2CLCs during investigations of the development of cloned embryos. The 2CLCs have similar transcriptome and chromatin features to that of 2-cell-stage embryos, and we propose that 2CLCs, already a valuable in vitro model for dissecting totipotency, will provide new opportunities to improve SCNT efficiency.

## 1. Introduction

Maternal factors in oocytes reset the parental DNA and histones across the genome of zygotes through epigenetic modification, which provides the preparation for genome-wide activation and establishment of totipotency. Upon fertilization, maternal factors trigger zygotic genome activation (ZGA), forming totipotent embryos with 2-cell-specific gene expression profiles. The main transcriptome of totipotent cells is characterized by the initiation of ZGA. Endogenous retrovirus elements, previously viewed as “deleterious genomic parasites”, are usually silenced in the vast majority of somatic cells by a combination of epigenetic modifications, such as DNA methylation or histone modifications, to minimize the risk of retrotransposition. In contrast, endogenous retrovirus elements, most notably *MERVL*, are transiently activated at the ZGA stage in mouse early embryos. Since a large number of 2-cell-specific transcripts use *MERVL* long terminal repeat (LTR) elements as alternative promoters, the *MERVL*-derived transcriptome constitutes an important component of ZGA, with *MERVL* activation regarded as a totipotent marker [1,2,3,4]. As development progresses, the *MERVL*-derived transcriptional program is rapidly shut off as early embryos exit the 2-cell stage, gradually losing their developmental potency. Only 2-cell-stage embryos in mice, or 4-/8-cell-stage embryos in other mammalian species, that enable a single cell to establish embryonic and extraembryonic tissues are characterized as totipotent [5,6,7,8,9,10].

Although embryonic stem cells (ESCs) have infinite proliferation potential and can differentiate into all three germ layers (i.e., endoderm, mesoderm and ectoderm), unlike 2-cell-stage embryos, they cannot differentiate into extraembryonic tissues [11]. Strikingly, recent studies have shown that ESCs exist as a heterogeneous population in culture, and 1% of mouse ESCs express *MERVL* and *MERVL*-driven 2-cell-specific genes. These totipotency-like cells, also known as 2-cell-like cells (2CLCs), exhibit expanded developmental potential beyond pluripotency, resembling 2-cell stage blastomeres [2].

Since the birth of Dolly in 1997 [12], the somatic cell nuclear transfer (SCNT) technique has been widely used and has great significance for animal breeding, biological medicine and the preservation of endangered species [13]. Although many improvements have been achieved in SCNT technology by regulating the epigenetic status of reconstructed embryos, SCNT-mediated reprogramming remains inefficient. It is well known that ZGA is incomplete, and 2-cell-specific genes are not properly activated in cloned embryos. This means that the establishment of totipotency often fails in cloned embryos, markedly impairing embryonic developmental potential [14,15,16,17].

Currently, little is known about the regulatory mechanisms underlying the establishment of totipotency in early cloned embryos. Understanding the principle behind the conversion from ESCs into totipotency-like cells is crucial for facilitating the establishment of totipotency. Investigations of totipotency require substantial amounts of sample input, limiting the application of various molecular and biochemical approaches used for studying the totipotency of mammalian embryos. Compared with 2-cell-stage embryos, which are difficult to obtain in large numbers, 2CLCs can be easily obtained and may provide a good model for investigating the establishment of totipotency and ZGA. Along with the similar gene expression patterns between 2CLCs and 2-cell-stage embryos, the strategies used for inducing the formation of 2CLCs may provide new opportunities for improvements in SCNT efficiency. This review aims to provide a snapshot of the practical strategies used to induce 2CLCs that may be applied to improve SCNT efficiency. This novel perspective on the study of totipotency in cloned embryos may contribute to the emergence of new approaches for improving SCNT efficiency.

## 2. Characterization of 2CLCs

Totipotency refers to the ability of a single cell to contribute to all embryonic germ layers, germline and extraembryonic tissues [18]. More strictly, only single cells that develop into a complete organism can be called totipotent cells [19,20]. With regard to mouse preimplantation embryos, totipotency is restricted to the 2-cell stage. Endogenous retroviruses are expressed in preimplantation embryos when ZGA is initiated. In particular, *MERVL* is transiently derepressed and produces 3% of the transcribed messenger RNA in 2-cell-stage mouse embryos [3,21,22]. The flanking LTR elements contain cis-regulatory sequences and RNA polymerase II promoters in *MERVL* control transcription initiation, and a large number of chimeric transcripts are generated [2,23]. Among them, over 90% are LTR–exon fusions that generate open reading frames, and then the *MERVL* 5′ LTR provides functional promoters for protein-coding genes; this indicates that *MERVL* activity is developmentally regulated, and the *MERVL* 5′ LTRs confer expression of 2-cell-specific genes. In view of the above facts, *MERVL* can be considered a marker for totipotent cells, and it may be feasible to use *MERVL* 5′ LTRs for labeling totipotency-like cells. Macfarlan et al. cloned and inserted the *MERVL* 5´ LTR and a portion of the gag gene upstream of the red fluorescent protein tandem dimeric Tomato, obtaining a 2C::tdTomato construct to monitor the expression of tdTomato in preimplantation embryos or ESCs in vitro. In line with theoretical expectations, tdTomato expression was highest in 2-cell-stage embryos and became downregulated at other developmental stages, demonstrating the efficiency of the 2C::tdTomato construct. Surprisingly, Macfarlan et al. also found that when the 2C::tdTomato construct was introduced into ESCs, 1% of ESCs were strongly labeled with tdTomato (denoted 2C::tdTomato^+^), with *MERVL* and 2-cell-specific genes expressed at the same time [2]. Importantly, like 2-cell-stage embryos, 2C::tdTomato^+^ ESCs lacked pluripotency markers, such as Oct4, Sox2 and Nanog, and contributed to the trophectoderm in addition to the inner cell mass (ICM) when injected into morula-stage embryos. Subsequently, 2C::tdTomato^+^ cells contributed to embryonic endoderm, ectoderm, mesoderm, the germ lineage, yolk sac and placenta during later development. These totipotency-like 2C::tdTomato^+^ ESCs were also called 2CLCs [2].

The 2CLCs share several similarities with 2-cell-stage embryos in various aspects, including developmental competence, transcriptomic features and chromatin organization. Like 2-cell-stage embryos, 2CLCs contribute to both embryonic and extraembryonic lineages, which display totipotency-like status and expanded cell fate potential [2,5]. The most prominent molecular features of totipotency are the expression of 2-cell-specific transcripts (including *Zscan4*, retrotransposons and major satellite repeats) and the downregulation of pluripotency genes (e.g., *Oct4*, *Sox2* and *Nanog*). Both 2-cell-stage embryos and 2CLCs display these transcriptomic features [2]. There is also similarity between 2-cell-stage embryos and 2CLCs in the histone mobility of chromatin. The assessment of histone mobility in ESCs and 2CLCs can be achieved through fluorescence recovery after photobleaching analysis of H3.1-GFP. Compared to ESCs, 2CLCs displayed a relatively high level of H3.1-GFP mobility, which was comparable to that of 2-cell-stage embryos [24,25]. Morphologically, compared with pluripotent and somatic cells, 2CLCs display major global differences in nuclear organization. Like zygotes and 2-cell-stage embryos, 2CLCs are characterized by a lack of chromocenters in the nucleus. It is known that DAPI-dense foci or the association of pericentromeric satellite DNA clusters are present in chromocenters in ESCs, whereas DAPI-rich regions in 2CLCs are spread out, without chromocenters [26]. Moreover, immature nucleoli structures and reduced rRNA output in 2CLCs are similar to nucleolar precursor bodies (NPBs) of 2-cell-stage embryos, with attenuated nucleolar function and distinct nucleolar components [27]. The 2CLCs also display highly relaxed chromatin architecture. That is, 2CLCs possess the similar open chromatin landscape to that of 2-cell-stage embryos, and chromatin accessibility at *MERVL* elements is increased in 2CLCs [26,28]. We propose that the relaxed chromatin architecture may enable transcription factors to access targets and permit the derepression of 2-cell-specific genes, retrotransposons and other repetitive sequences wrapped in heterochromatin structures. Recently, it was discovered that through chromatin conformation capture protocols, assessment of long-range chromatin interactions, such as topologically associating domains (TADs) and loops, can be achieved. This research has shown that TADs and loops are weaker in 2CLCs than in ESCs, and similar rearrangement of the chromatin architecture also occurred in early embryos [29,30]. Beyond that, widespread demethylation also takes place across the genome after ESCs enter the 2-cell-like status, especially in *MERVL* elements [31]. A similar dynamic DNA demethylation also occurs in early mouse embryos [32]. Interestingly, 2CLCs and 2-cell-stage embryos share similar metabolic characteristics. For example, because of a lower mitochondrial respiratory capacity, 2CLCs exhibit more reduced oxygen consumption rates than those of ESCs and resemble those of early embryos [33,34]. Hu et al. observed decreased glycolytic activity in 2CLCs, consistent with that observed in 2-cell embryos [35,36]. The similarities between 2CLCs and 2-cell-stage embryos are summarized in Table 1.

## 3. The Strategies Used for Inducing 2CLCs May Improve SCNT Efficiency

According to stringent criteria, 2CLCs may not be totipotent because a single 2-cell-like cell cannot develop into a complete organism. However, this kind of cell provides a novel way to study certain aspects of totipotency. It has been shown that 2CLCs share many similarities with the blastomeres in 2-cell embryos, and 2CLCs display a better reprogramming ability when used as nuclear donors during the SCNT process [26]. To date, the regulatory mechanisms controlling totipotent status in cloned embryos remain elusive, and placental dysfunction is widespread in cloned animals, possibly reflecting a deficiency of totipotency establishment in cloned embryos. Therefore, 2CLCs may provide valuable in vitro models to dissect common regulators or pathways in totipotency regulation. More importantly, the strategies that convert ESCs into 2CLCs have provided a new opportunity to improve SCNT efficiency, as described in the following text.

### 3.1. Effect of Regulating Histone-Modifying Enzymes on the Developmental Competence of Early Cloned Embryos

Repressive histone modification limits the broad pattern of transcription in 2-cell-stage embryos [37,38]. Through in situ hybridization and immunofluorescence microscopy, Macfarlan et al. demonstrated that the level of *MERVL* and several 2-cell-specific genes were significantly upregulated in mouse ESCs with mutated lysine-specific demethylase 1A (KDM1A) and histone lysine methyltransferase of H3K9 (G9a) [2]. KDM1A and G9a are both involved in the repression of 2-cell-specific genes and the transition between ESCs and 2CLCs; therefore, inhibiting associated repressive histone modification may also be beneficial for SCNT efficiency. By treating donor cells with 2-phenylcyclopropylamine (a specific KDM1A inhibitor), the level of H3K4me2 in cloned goat embryos increased markedly compared with the control group [39]. The aberrant methylation status of H3K9me and H3K9me2 also exists in cloned embryos. Following activation, cloned porcine embryos treated with 50 nm BIX-01294 (an inhibitor of G9a) for 14–16 h had decreased levels of H3K9me2 and H3K9me, and the rate of blastocyst formation was increased. More importantly, BIX-01294-treated cloned embryos could develop to term with a higher cloning efficiency than the control group [40].

Treatment of 2C::tdTomato ES lines (using *MERVL* elements to label 2-cell embryos) with trichostatin A (TSA), an inhibitor of histone deacetylase, yielded a four-fold increase in the number of 2C::tdTomato^+^ cells (cells with 2-cell-embryo-like characteristics) [2]. TSA also enhances the pool of acetylated histones and DNA demethylation [41], implying that the developmental potential of cloned embryos may benefit from higher reprogramming efficiency induced by TSA. Currently, TSA is widely used to increase the overall efficiency of SCNT. For instance, following activation, cloned porcine embryos treated with 50 nM TSA exhibited an increased rate of blastocyst formation compared with controls (46.4% versus 17.7%, respectively) [42]. Likewise, Rybouchkin et al. observed a higher blastocyst rate from TSA-treated cloned mouse embryos [41].

### 3.2. Effect of Regulating Methylation Levels on the Developmental Competence of Early Cloned Embryos

The conversion from ESCs into 2CLCs was characterized by a two-step transcriptional reprogramming process, including downregulation of pluripotent genes in the first step and upregulation of the 2-cell-specific genes in the second step. Through a CRISPR–Cas9-mediated screen, Fu et al. revealed that Myc hampers downregulation of pluripotent genes during the ESC to intermediate-state transition, while DNMT1 hinders 2-cell-specific genes expression during the intermediate-to-2CLCs transition. Fu et al. further demonstrated that DNMT1 deficiency significantly increased the 2CLCs population in ESC clones [43]. Mechanistically, degradation of UHRF1 and DNMT1, caused by the transient burst of Zscan4, may be involved in global DNA demethylation in 2CLCs [44]. Incomplete DNA methylation reprogramming leads to the inefficient activation of genes essential for the embryonic development, then causing low development of cloned embryos finally [45]. Transcript degradation of DNMT1 in porcine cloned embryos are delayed compared with that of their fertilized counterparts, which may impede the demethylation reprogramming of cloned embryos. After injecting anti-DNMT1 siRNA (50 μmol/L) into cloned embryos at 1-cell stage, the rate of blastocyst formation increased from 25.74% to 30.43% [46]. It is well known that DNMT1s (i.e., the isoform of DNMT1 caused by alternative usage of multiple first exons) are usually expressed in donor somatic cells; thus, cloned embryos inevitably contain the unwanted DNMT1s before ZGA. Correspondingly, when DNMT1s in donor cells were removed prior to SCNT, more cloned embryos finished the ZGA procedure, and the rate of blastocyst formation was increased significantly [47].

### 3.3. Effect of Double Homeobox (DUX, i.e., Pioneer Transcription Factors) on the Developmental Competence of Early Cloned Embryos

Double homeobox (DUX, in mouse) or DUX4 (in humans) directly bind to *MERVL* or 2-cell-specific genes promoters and have been viewed as potent *MERVL*/*HERVL* and 2-cell-specific gene activators that can convert ESCs to a 2-cell-like status [28,48,49]. DUX is transiently expressed in a specific time window (i.e., early 2-cell stage) and is not activated in cloned 2-cell embryos; thus, DUX target genes, such as the *Zscan4* family and *MERVL*, are silenced [50]. When DUX was overexpressed in mouse SCNT embryos, the rate of 2-cell block decreased from 54.7% to 9.6% [51], whereas prolonged DUX overexpression in ESCs caused DNA damage and apoptosis [52]. Therefore, precise regulation of the overexpression of DUX may be beneficial for improving the development of cloned embryos. To accurately regulate the overexpression of DUX, transgenic mouse lines containing a doxycycline (dox)-inducible *DUX* gene were generated. Yang et al. found that when transgenic cloned embryos were treated with dox for 24 h after 11 h activation, the blastocyst formation rate of cloned embryos was significantly increased, and switching between a dox-containing and dox-free culture medium at different time periods indicated that the beneficial effect of DUX on cloned embryonic development was time-dependent [53]. Furthermore, single-embryo RT-qPCR and immunofluorescence results showed that the overexpression of DUX increased the expression of 2-cell-specific genes such as *MERVL* and *Zscan4* [51]. The H3K9ac signal in cloned embryos was much lower than that in fertilized embryos. Aberrantly acetylated regions in cloned embryos provide epigenetic barriers, and DUX can recruit the histone acetyltransferases EP300 and CREB binding protein to its target sites and induce chromatin relaxation and transcriptional activation of nearby genes. Yang et al. even found that overexpression of DUX increased the H3K9ac signals in promoters of 2-cell-specific genes (target genes of DUX), and corrected the aberrant H3K9ac signal in cloned embryos, ultimately leading to appropriate ZGA and improved SCNT efficiency [50].

### 3.4. Effect of Regulating Reorganization of 3D Chromatin Structure on the Developmental Competence of Early Cloned Embryos

Cohesin, an architectural protein complex, works with CCCTC-binding factor (CTCF) to play a key role in TAD formation [54,55]. Using auxin-induced degron to deplete sister chromatid cohesin 1, a key subunit of cohesin in mouse ESCs, it was unveiled that minor ZGA genes, such as *Zscan4a/b*, were derepressed after auxin treatment, implying that these totipotency-associated genes are targets of cohesin [56,57]. Using low-input, high-throughput chromosome conformation capture techniques, Zhang et al. showed that the reorganization of the 3D chromatin structure was abnormal in cloned embryos, and these embryos show stronger TADs at the 1-cell stage than that of fertilized embryos, indicating that chromatin relaxation was less efficient in cloned embryos than in fertilized embryos [58]. Because of the resistance to reprogramming, the TAD boundary may cause misregulated expression of nearby genes in cloned embryos. It remains an open question whether the decrease in TADs and boundary insulation may promote cloned embryo development. Through removing cohesin in donor cells, Zhang et al. further revealed that these cloned embryos had increased blastocyst rates, and some minor ZGA genes, such as *Zscan4*, that were previously silenced in cloned embryos were activated [58].

### 3.5. Effect of Regulating Chromatin Assembly on the Developmental Competence of Early Cloned Embryos

Chromatin assembly factor 1 (CAF-1), a trimeric complex, is responsible for the deposition of histones H3 and H4 during DNA synthesis [59,60]. Previous research has shown that depletion of the p150 subunit of CAF-1 results in pericentric heterochromatin instability in Drosophila [61]. Recently, Ishiuchi also revealed that mouse ESCs can be induced into 2CLCs in vitro via downregulation of the p150 or p60 subunits of CAF-1, with chromocenters lost and cells exhibiting similar transcriptional profiles to that of 2-cell-stage embryos. In particular, totipotent marker genes, such as 2-cell-specific genes, MERVL and major satellites, were significantly upregulated in these 2CLCs. The positive effect upon *MERVL* expression was attributed to changes of chromatin accessibility caused by CAF-1 knockdown. Using micrococcal nuclease digestion, Ishiuchi further revealed that after p60 was knocked down in ESCs, the global accessibility to micrococcal nuclease was higher in 2CLCs, and *MERVL* LTRs were indeed more accessible [26]. In relation to this, we raise the question of whether regulating nuclear organization via inhibition of CAF-1 could improve SCNT efficiency. Next, Ishiuchi’s research showed that since the reprogramming potential of donor cells depended on the potency state of the donor nucleus [62], the rate of blastocyst formation of cloned embryos derived from p60 knockdown-induced totipotency-like cells was 2.5-fold higher than that of cloned embryos derived from ESCs [26]. The genomic areas refractory to reprogramming have been identified, named as reprogramming-resistant regions (RRRs), and cloned embryos often fail to be completely reprogrammed because of RRRs [15]. Surprisingly, analysis of RRRs revealed that a large proportion of genes, which were transcribed in normal 2-cell fertilized embryos but not in cloned embryos, were significantly upregulated in p60-knockdown and p150-knockdown-induced 2CLCs. These findings indicated that, as totipotent embryos are better nucleus donor cells for SCNT, regulating chromatin assembly via p60- or p150-knockdown could induce 2CLCs, and these induced 2CLCs may have a higher reprogramming competency than ESCs [26].

### 3.6. Effect of Regulating rRNA Synthesis and Nucleolar Maturation on the Developmental Competence of Early Cloned Embryos

**Figure 1:**
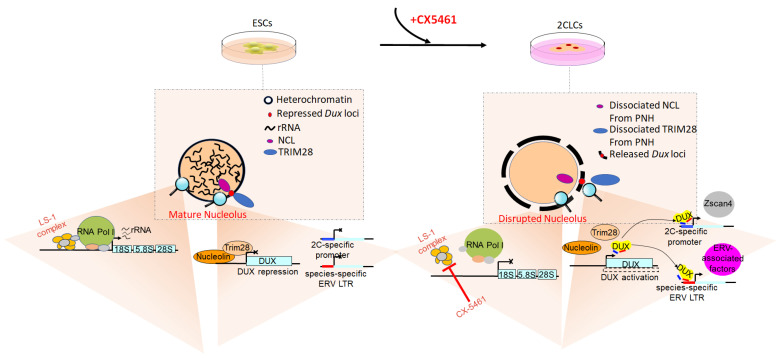
Scheme of cell fate conversion via rRNA biogenesis. In the mature nucleoli of embryonic stem cells (ESCs), high-abundance rRNA maintains nucleolar liquid–liquid phase separation (LLPS) and integrity, thus facilitating nucleolin (NCL)/tripartite motif-containing 28 (TRIM28) complex occupancy on *DUX* loci, causing *DUX* repression in perinucleolar heterochromatin. ESCs do not display transcriptome features similar to those of 2-cell-stage embryos, especially without DUX activation. In contrast, in the disrupted nucleolus of 2-cell-like cells (2CLCs), adding CX-5461 inhibits the recruitment of the selective factor 1 (SL-1) complex and polymerase I (Pol I) initiation factor to rDNA, thus impairing rRNA synthesis. The inhibition of rRNA synthesis interferes with nucleolar LLPS and then disrupts nucleolar integrity and causes dissociation of the NCL/TRIM28 complex from *DUX* loci, providing a permissive environment for *DUX* derepression. Furthermore, DUX functions as a core transcription factor for zygotic genome activation (ZGA) and drives the expression of *MERVL* and 2-cell-specific genes (e.g., *Zscan4*), which allow 2CLCs to obtain transcriptome features that resemble those of 2-cell-stage embryos.

There are intriguing links between rRNA synthesis, nucleolar maturation and gene expression during the development of early embryos. Originally revealed by Percharde’s research, nucleolin (NCL, the major subcompartment of the nucleolus) functions as a DUX repressor, indicating that the nucleolus may play a potential role in 2-cell exit [63]. Functions of the nucleolus go beyond ribosome biogenesis. The nucleolus and rRNA biogenesis are also involved in the location and regulation of repressive genomic domains and then establish a hub for the organization of inactive heterochromatin in the cell [64]. Xie et al. found that nucleolar disruption was sufficient to release DUX and induce ESCs into 2CLCs; furthermore, it was shown that nucleolar disruption also leads to DUX activation and 2- to 4-cell arrest in preimplantation embryos [27]. Precisely how disrupting the nucleolus converts ESCs into the 2-cell status has been the topic of many investigations. It is well known that upon fertilization, early totipotent embryos (i.e., zygotes or early 2-cell embryos) possess immature nucleoli, also known as NPBs [65]. The NPBs lack RNA and strong staining for NCL protein, which only becomes detectable in surrounding nucleoli at the late 2-cell stage, when 2-cell-specific genes are shut down [27]. Detectable amounts of RNA appear on the NPB surface only at the end of the 2-cell stage, when rRNA gene transcription resumes [66]. Morphologically, compared with the mature nucleoli in ESCs, the immature nucleoli in 2CLCs are more similar to NPBs exclusively found in early totipotent embryos. Xie et al. also found a significant reduction in nascent RNA synthesis, including nucleolar rRNA, in 2CLCs [27]. It can be said that early totipotent embryos and 2CLCs are characterized by the presence of immature nucleoli. The question remains whether disrupted nucleolar and attenuated nucleolar function could drive the 2-cell-like state. Recently, Xie et al. inhibited RNA polymerase I (Pol I) and rRNA synthesis with CX-5461 and found that nucleoli were disrupted, and the expression of 2-cell-specific genes and *MERVL* was upregulated in ESCs. Of particular note is that DUX, usually regarded as a *MERVL* and 2-cell-like gene activator, was also upregulated following nucleolar disruption [27]. The reasons for this may be as follows. *DUX* is usually attached in repressive perinucleolar heterochromatin, and the liquid–liquid phase separation (LLPS) properties of the nucleolus, which rely on the activity of RNA Pol I, maintains this spatial configuration. Once rRNA transcription is disturbed or phase separation is inhibited, nucleolar integrity is inhibited, and *DUX* is dissociated from the nucleolar periphery and derepressed. Ultimately, nucleolar disruption is sufficient to convert ESCs into 2CLCs [27]. Mechanistically, rRNA participates in maintaining the LLPS of the nucleolus and the establishment of perinucleolar heterochromatin, and then the LLPS facilitates the NCL/tripartite motif-containing 28 protein (TRIM28) complex localization on the *DUX* locus, eventually repressing DUX expression [67,68,69]. Yu et al. found that inhibiting rRNA biogenesis via CX-5461 treatment disrupted the normal liquid-like phase of the nucleolus and caused dissociation of the NCL/TRIM28 complex from perinucleolar heterochromatin [69], as shown in Figure 1. Previous studies showed that phase separation can regulate the epigenetic state of chromatin [70,71,72]. In line with these studies, Yu et al. also found that the levels of H3K9me3 and H3K27me3 were lower, while the levels of H3K4me3 and H3K27ac were higher, at nucleolar-associated domains when ESCs were treated with CX-5461. At the same time, similar epigenetic changes appeared around the *DUX* locus [69].

The NPBs in pseudo-pronuclei of cloned embryos exhibit abnormalities and may cause the different developmental potential observed between cloned and fertilized embryos [73,74]. Recent research has also demonstrated that transiently inhibiting rDNA transcription via CX-5461 in donor somatic cells can improve the preimplantation development of cloned embryos [75]. As in the vast majority of somatic cells, *DUX* loci in mouse embryonic fibroblasts (i.e., the donor cells for SCNT) were found to be attached to the nucleolar periphery enriched for repressive histone marks; thus, DUX often fails to be reactivated during reprogramming in early cloned embryos [15,76,77]. Recently, Liao et al. found that following treatment with CX-5461, upstream binding factor (UBF) fluorescence intensity was less, and the number of black silver particles declined in the cumulus cells, indicating that CX-5461 treatment inhibited the activity of rDNA and then accelerated the deterioration of the mature nucleolus [75]. Using CX-5461-treated embryonic fibroblasts as donor cells, further SCNT revealed that, compared with the control group, the early cloned embryos contained a more unconsolidated reticular-like ultrastructure in the nucleoli, resembling NPBs in early fertilized embryos. Importantly, the rate of blastocyst formation increased from 24% to 34% [75]. Taken together, we speculated that CX-5461 accelerated nucleolar degeneration and enhanced the conversion of matured nucleoli of donor somatic cells into NPBs that are exclusive to early totipotent embryos, thus leading to improvement in the preimplantation development of the treated cloned embryos.

### 3.7. Effect of Regulating Replication Features on the Developmental Competence of Early Cloned Embryos

The remodeling of replication features also participates in cell fate control and reprogramming. Through DNA fiber analysis for measuring replication fork speed [78,79], Nakatani et al. revealed that fork speed was 1.34 kb min^−1^ in ESCs and 0.56 kb min^−1^ in 2CLCs. Moreover, 2CLCs used more origins of replication than ESCs to compensate for a slower fork speed due to the unchanged length of the S phase. In addition, totipotent 2-cell stage mouse embryos recapitulated this feature of the replication fork in 2CLCs, indicating that slow replication dynamics are also a hallmark signature of totipotent cells, and manipulating replication fork speed may regulate reprogramming towards a totipotency-like status [80]. It is known that under the influence of hydroxyurea, an antineoplastic agent that inhibits deoxyribonucleic acid synthesis, cells released synchronously into S phase undergo a 10-fold reduction in replication fork speed [81]. Nakatani et al. used low doses of hydroxyurea to slow down the replication fork speed in ESCs. As expected, the replication fork speed was slowed down by hydroxyurea treatment, concomitant with a 10-fold increase in totipotency-like cells that displayed typical 2CLCs features. Nakatani et al. considered that regulating replication fork speed may also be applicable to reprogramming of the induced pluripotent cell (iPSC), shown by the increase in iPSC colonies after exposure to hydroxyurea [80]. Therefore, it is natural to wonder whether regulating replication fork speed has a conserved role in cell reprogramming, and it could even be applicable to reprogramming of SCNT. Nakatani et al. found that reducing fork speed improved SCNT efficiency. Using cumulus cells as donors for SCNT, the reconstructed cloned embryos were treated with 10 μM hydroxyurea for 24 h after activation. Hydroxyurea treatment induced a 3.5-fold increase in the rate of blastocyst formation compared with the control group. Remarkably, through principal component analysis following single-embryo RNA sequencing, it was shown that ZGA-associated genes were efficiently activated in hydroxyurea-treated cloned embryos. The transcript expression levels across RRRs were higher in hydroxyurea-treated cloned embryos than those in cumulus cells or control cloned embryos. Thus, these results implied that slowing replication fork speed via hydroxyurea treatment can facilitate reprogramming, ultimately improving the developmental potential of cloned embryos [80].

Aphidicolin (APH) has also been identified as a positive regulator of 2CLCs. Recently, Atashpaz et al. revealed that the ataxia telangiectasia and Rad3-related kinase (ATR)-mediated response to replication stress can induce the establishment of a 2-cell status in mouse ESCs and embryos. The ATR-induced conversion into 2-cell-like status was carried out via post-transcriptional regulation of DUX. The G-rich sequence factor 1 protein directly binds to *DUX* mRNA to promote its stability, which allows ATR to indirectly control DUX [82]. APH, acting as a reversible inhibitor of DNA polymerases, can activate ATR by stalling replication fork progression [83]. Therefore, APH treatment may result in a global transcriptional activation of 2-cell-specific genes. Indeed, following APH treatment, the identified differentially expressed genes overlapped with genes specifically expressed in 2CLCs, including *MERVL*, *MT2_Mm*, *DUX*, *Eif1a*-like genes (*Gm5662, Gm2022, Gm4027* and *Gm8300*), *Zscan4* genes (*Zscan4b* and *Zscan4d*), *Zfp352*, *Zfp750*, *Tdpoz* genes (*Tdpoz1* and *Tdpoz3*) and *Tmem92* [83]. Interestingly, in 2012, Zhang et al. found that, prior to carrying out SCNT, treating donor somatic cells with APH improved the developmental potential of cloned embryos. For APH treatment, donor somatic cells were cultured under serum starvation for 2 days, then treated with 0.1 mg/mL APH for 1 day. Immediately following the SCNT procedure, Zhang et al. observed significantly elevated blastocyst formation rates in the APH-treated group [84]. There is general agreement that cycle synchronization of donor and recipient cells is one of the major factors that affect SCNT efficiency [12,85]. Accordingly, Zhang et al. demonstrated that serum starvation plus APH treatment synchronized donor cells at the pre-S phase and improved the developmental potential of cloned embryos [84]. Here, we propose that synchronized donor cells are indeed beneficial for SCNT, and APH, as a positive regulator of 2CLCs, may induce the donor cells toward some degree of totipotency-like status, which has been implied by transcriptomic changes following APH treatment [82].

### 3.8. Effect of Regulating Energy Substrates on the Developmental Competence of Early Cloned Embryos

It was recently revealed that lactate and lactate-induced histone lactylation are also involved in cell fate conversion in mouse ESCs [86]. Exogenous lactate stimulates the lactylation of histone lysine residues [87], and lactate treatment for 24 h significantly improved lactylation of H3K18 (H3K18la) in ESCs [86]. Findings from chromatin immunoprecipitation-sequencing and RNA-sequencing analysis of mouse ESCs found a positive correlation between H3K18la intensity and transcript abundance. For example, a significant upregulation of ZGA marker genes, such as *Zscan4*, *Zfp352*, *Tcstv3* and *Sp110*, was observed, consistent with previous results that lactate raised the ratio of 2-cell-like ESCs [33,86]. To further dissect the regulatory mechanisms underlying ZGA gene activation, Tian et al. investigated the occupancy landscape of H3K18la and RNA polymerase II (Pol II) upon lactate supplementation and found increased deposition of H3K18la at the transcription start site or gene body of upregulated genes. In addition, Pol II occupancy at the gene body also increased. Tian et al. proposed that H3K18la may recruit p300, a transcription coactivator, to facilitate transcription elongation, and Pol II elongation derived from histone lactylation may be achieved by loosening nucleosome–DNA interactions and forming a platform for coactivator binding [87]. Taken together, the above findings indicated that lactate resulted in global upregulation of ZGA genes, which may trigger other downstream events, especially in preimplantation embryos. Therefore, lactate may also regulate the preimplantation embryonic development of cloned embryos. In 2003, it was found that supplementing the culture medium with lactate/pyruvate improved the preimplantation developmental competence of cloned porcine embryos. When cloned porcine embryos were cultured in an NCSU-23 medium supplemented with lactate (5.0 mM)/pyruvate (0.5 mM), the cleavage and blastocyst formation rates were significantly higher than those of the control group [88]. Pyruvate is necessary for mitochondrial respiration and cell cleavage during preimplantation development, and lactate in the culture medium can facilitate the absorption and metabolism of pyruvate [89,90]. Lee et al. considered that it was the above properties of lactate that improved the development of cloned embryos [88]. Given the strong contributory role played by lactate in cell fate control of mouse ESCs, we speculate that lactate may also enhance the activation of ZGA genes by stimulating the lactylation of histone lysine residues, although this proposal requires further investigation.

### 3.9. Other Strategies That Have Not Been Applied to Improve SCNT Efficiency

Several gatekeepers also prevent ESCs from entering a 2-cell-like status. For instance, CTCF has been identified as a barrier for entering the 2-cell-like status. The expression level of 2-cell-specific genes, such as *MERVL* and *Zscan4*, increased following CTCF depletion, with restoration of CTCF levels facilitating the exit from a 2-cell-like status [52]. Interestingly, Olbrich et al. found that DNA damage induced by DUX was localized to CTCF binding sites. Thus, DUX-induced DNA damage may be associated with single or double strand breaks at CTCF sites. Totipotent zygotes or blastomeres of early 2-cell embryos are characterized by weak TADs and a relaxed chromatin state. CTCF, a zinc-finger binding protein, is involved in chromosome folding and the insulation of TADs [91], and then the transition from totipotency to pluripotency is marked by the accumulation of CTCF and maturation of TADs in early embryos [29,92,93]. Therefore, depletion of CTCF in ESCs leads to the establishment of totipotent status [52].

Expanded stem cell potency can also be obtained by the activation of miR-344. Zinc-finger MYM-type containing 2 protein (Zmym2) recruits a lysine-specific demethylase 1 sd1/histone deacetylase corepressor complex to *MERVL* LTRs and inhibits the expression of *MERVL*-associated genes. MiR-344, the target of DUX, can negatively control the expression of Zmym2 at the post-transcriptional level. Ultimately, miR-344-2 alone is sufficient to release the repressed *MERVL*-associated genes and convert ESCs into a totipotency-like status [94].

The spliceosome complex also controls the transition from totipotency to pluripotency. Knockdown experiments of different splicing factors (including Snrpd2, Snrpb, Isy1, Eftud2, Lsm4, Puf60, Sf3b2 and Sf3b5) in ESCs indicated that totipotent marker genes such as *Zscans* and *MERVL* were upregulated, while pluripotent marker genes such as *Pou5f1*, *Nanog* and *Sox2* were downregulated. By adding pladienolide B, a splicing inhibitor that specially inhibits the function of spliceosomes, to the culture medium for mouse ESCs, Shen et al. further showed that merely employing a specific spliceosome inhibitor may be sufficient to induce a totipotency-like status [95,96]. Mechanistically, when pladienolide B was added into the ESC culture medium, with fewer and shorter introns, the totipotency-associated genes were efficiently spliced and activated, while the splicing of pluripotency-associated genes was inhibited [96]. 

Although the above strategies have successfully converted ESCs into totipotency-like cells, experimental dissection of the function of these strategies has not been performed for cloned embryos. Further investigation should determine the efficiency of the above strategies in the establishment of totipotency in cloned embryos.

## 4. Conclusions

**Figure 2:**
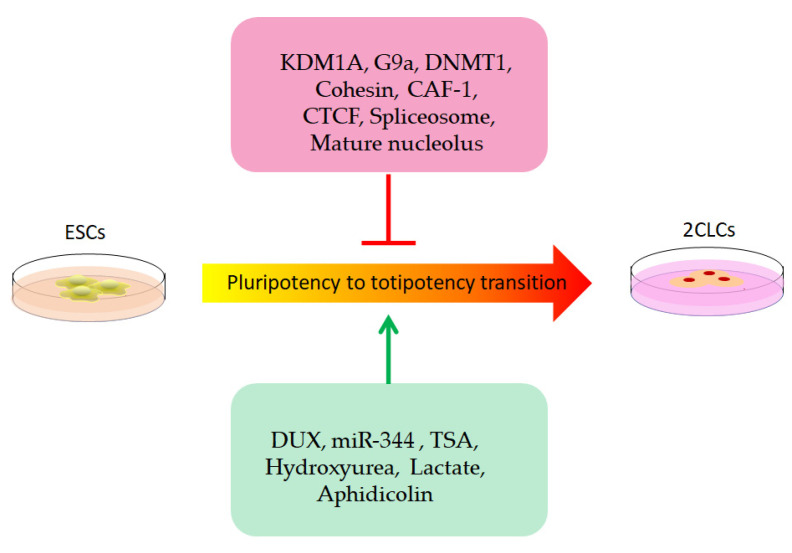
Schematic figure summarizing factors that promote or inhibit the transition from ESCs to 2CLCs. On the one hand, inhibitors, such as KDM1A, G9a, DNMT1, cohesin, CAF-1, CTCF, spliceosome and mature nucleolus, impede 2CLCs formation; on the other hand, some cellular active small molecule or chemicals, such as DUX, miR-344, TSA, hydroxyurea, lactate and aphidicolin, drive pluripotency to totipotency transition and facilitate 2CLCs formation.

Taken together, we summarized factors that promote or inhibit the transition from ESCs to 2CLCs, as shown in Figure 2. Since the identification of 2CLCs, a new era has begun in terms of understanding the mechanisms of totipotency. Fortunately, many strategies that convert ESCs into 2CLCs have also been applied in facilitating the embryonic development of cloned embryos and may provide a novel perspective for improving SCNT efficiency. We should also bear in mind that not all strategies that convert ESCs into 2CLCs will be applicable for the establishment of totipotency in cloned embryos. With the popularity of high-throughput bioinformatic tools, future investigations should further identify the critical molecules or epigenetic signatures associated with totipotency and ultimately focus on common regulators or pathways in totipotency regulation. Such investigations will provide more insights into the mechanisms underlying the establishment of totipotency in cloned embryos and ultimately provide potentially novel approaches for improving SCNT efficiency.

## Figures and Tables

**Table 1:** Known similar features of 2CLCs as 2-cell-stage embryos.

Features	2-Cell-Stage Embryo	2CLCs	Reference
Developmental competence	totipotent	expanded cell fate potential	[2,5]
2-cell-specific transcripts	high level	high level	[2]
Pluripotency genes	downregulation	downregulation	[2]
Histones mobility	high core-histone mobility (H3.1, H3.2 and H2A)	high core-histone mobility (histones H3.1 and H2A)	[24,25]
Chromocenters	no defined chromocenters	no defined chromocenters	[26]
Nucleolus formation	NPBs	ring-like immature nucleoli	[27]
Chromatin accessibility	open chromatin landscape	open chromatin landscape	[26,28]
Higher-order chromatin organization	TADs and loops are weak	TADs and loops are weak	[29,30]
Global DNA methylation	global hypomethylation	global hypomethylation	[31,32]
Oxygen consumption	low	low	[33,34]
Glycolytic activity	low	low	[35,36]

## Data Availability

Not applicable.
